# Curved trajectories in stereotactic neurosurgery: is it feasible?

**DOI:** 10.1007/s10143-025-03976-2

**Published:** 2025-12-18

**Authors:** Dörthe Keiner, Julian Mühlenhoff, Mohamed Henia, Fabian Rabel, Wolfgang Reith, Matthias K. Hoffmann, Kathrin Flaßkamp, Karl Worthmann, Thomas Sattel, Joachim Oertel

**Affiliations:** 1 Klinik für Neurochirurgie, Universitätsklinikum des Saarlandes und Medizinische Fakultät, Universität des Saarlandes, Homburg/Saar, 66421 Germany; 2 Mechatronics Group, Technische Universität Ilmenau, Ilmenau, Germany; 3 Systems Modeling and Simulation, Saarland University, Saarbrücken, Germany; 4 Klinik für diagnostische und interventionelle Neuroradiologe, Universitätsklinikum des Saarlandes und Medizinische Fakultät der Universität des Saarlandes, Homburg, Germany

**Keywords:** Stereotactic neurosurgery, Curved trajectory, Intracranial stereotactic localization, Concentric tube continuum robot, Actuation system

## Abstract

**Objective:**

Stereotactic procedures are planned and performed with straight trajectories. However, brain sulci with vessels and ventricles have to be avoided for bleeding and deviation risks. In distinct pathologies such as insular or pineal lesions, or in patients with brain atrophy, curved trajectories theoratically could provide superior results. So far, research on curved trajectories for stereotactic neurosurgery focusses on aspects like path planning, robot design and control. In a collaborative project of engineers, mathematicians, and neurosurgeons a prototype system for curved cannulas was developed using a concentric tube continuum robot (CTCR) platform.

**Methods:**

Target precision and follow-the-leader-deviations by movements of the cannulas were assessed. For a set of automatically planned configurations by numerical optimization, the real robot behavior was compared to state-of-the-art models of elastostatic behavior. Target accuracy was tested with CT-imaging of a head model and a deep-seated target, calculation and transformation of stereotactic coordinates to the actuation system, and application of the curved cannulas to the target.

**Results:**

Determination of the model’s target point via CT-scan and transformation of the stereotactic coordinates in the path planning software for curved trajectories were possible. The technical operation of the prototype was improved for mounting at the stereotactic system. The target could be calculated with optimal accuracy in the panned configuration by numerical optimization and the test procedure could be performed successfully. However, accuracy with curved cannulas in this prototype system had a target point deviation of 2 mm and more.

**Conclusions:**

To the authors’ knowledge, this is the first application of curved cannulas for stereotactic neurosurgery in a comparable way to clinical practice. Further research will have to address incorporating iterative learning control of the robot’s tip position to reach target point deviations below 1 mm.

## Introduction

Stereotactic procedures are planned and performed with highly precise instruments especially designed for deep-seated and small structures. Since the 1950 s, these procedures have been used for minimally invasive biopsies, to perform thermal ablation in movement disorders, and to implant seeds for intracranial brachytherapy. Over the past three decades, an increasing number of patients has been treated with deep-brain stimulation (DBS) for movement and psychiatric disorders.

**Fig. 1:**
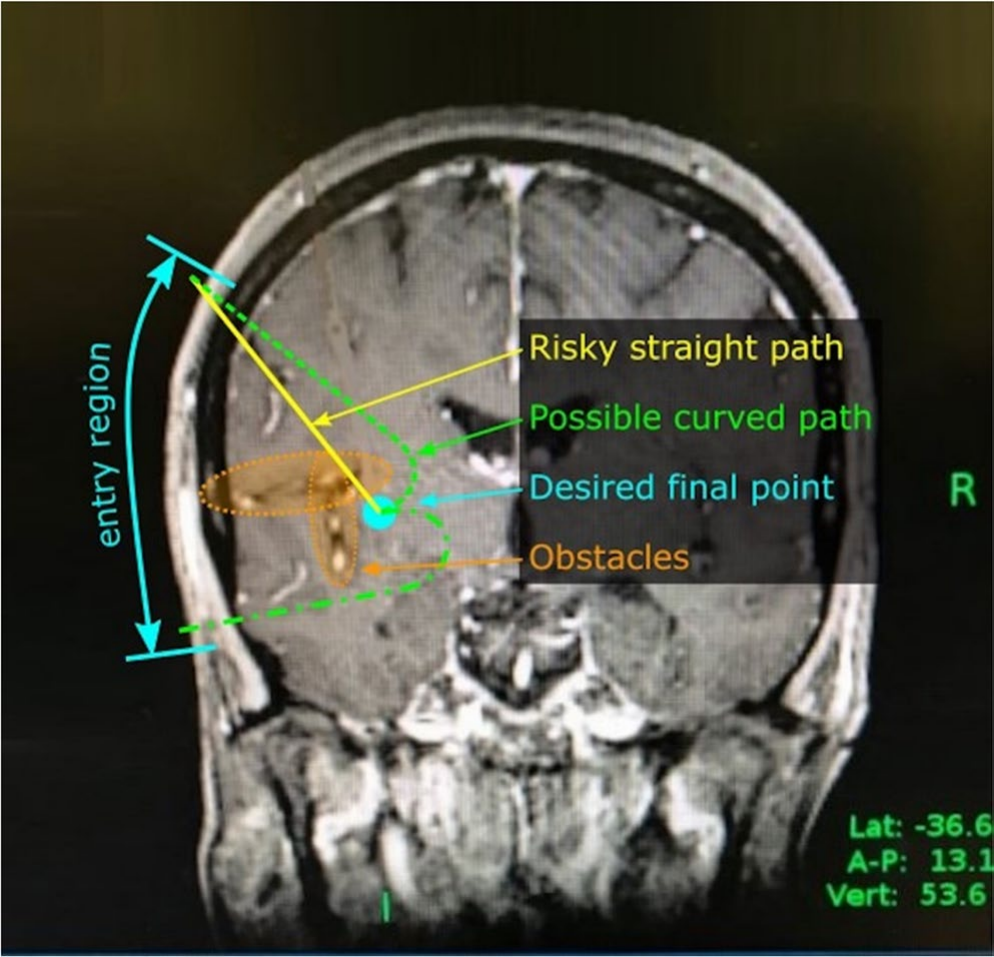
Comparison of straight and curved trajectory towards a lesion of the insular region. Possible trajectories to an insular lesion with curved cannulas (green path). In contrast, a straight trajectory (yellow path) would carry the risk of vascular injury or trajectory inaccuracy due to possible distortion of the cannula when passing the sulci

In daily clinical practice, stereotactic procedures require meticulous path planning with the help of medical software that allows preoperative viewing, manipulating and target-planning of the individual patients’ MR-images and CT-scans. This allows an individualized surgical approach while minimizing the risk of neurological damage. Deep-seated and small structures require high accuracy with deviation below 1 mm. So far, intracranial stereotactic procedures are limited to straight cannulas. This might cause problems and show limitation if brain sulci or ventricles have to be crossed because of risk of vessel injuries and inaccurate trajectory deviations. Thus, the use of curved paths might open up possibilities for additional surgical scenarios and a significant reduction of the surgical risks (Fig. 1).

Over the past years, cannulas with actively adjustable curvatures have been proposed for different disciplines such as neurosurgery, cardiac surgery, and urology focusing on general minimally invasive surgical techniques [1–4]. Only a few engineering research groups are systematically working on the development of curved cannula robots specifically designed for neurosurgical treatment using novel and innovative instruments [5–7]. Regarding neurosurgical procedures, research groups focused on techniques for the potential application in epilepsy surgery [5, 8] and intracranial endoscopy [9]. The application of an “intracranial minirobot” with curved pathway for brain tumor diagnosis and therapy has also been introduced [7].

From a mathematical point of view, optimal path-planning for stereotactic neurosurgical procedures is highly challenging. Several study groups focused on the use of concentric tube robots (CTCRs) to form curved trajectories with high precision for medical applications [5, 10–14]. CTCRs are comprised of two or more pre-curved and elastically deformable tubes with different diameters that are concentrically assembled. All tubes are actuated translationally and rotationally, allowing for continuous pathways being established by the robot, similar to an elephant’s trunk. Combined, the tubes are extended and retracted in different translational and rotational directions depending on the predefined curvature of the tube. In the past years, several general kinematic models for CTCRs have been developed incorporating bending and torsion, and torsional interaction among tubes. Emphasis was on actuation and predicted shape of cannulas. Findings were verified in numerical case studies on brain surgery [15]. Further, Leibrand et al. developed a software for interactive path planning and intraoperative surgical control for CTCRs [13].

Although CTCRs were investigated extensively in the context of mathematics and engineering, so far research has not resembled surgical procedures and resulting demands on these systems. Regarding stereotactic neurosurgery, several aspects are important:

- Is the application of curved trajectories to target intracranial pathologies with high precision technically possible?
- Which properties should curved cannulas have?
- How is the traumatic impact on surrounding brain tissue due to inevitable lateral movements during extension and retraction of the cannulas?
- How can a CTCR system be implemented into a neurosurgical stereotactic procedure regarding hardware and software demands of the OR’s armamentarium?

For investigations resembling stereotactic neurosurgical procedures, the following hardware and software components are required:

- 3D cranial phantom for CT- and MRI-scans including one or more target point(s).
- Cranial CT- and MR-image data set.
- Software system for integration of images for manual and automatic path planning based on entry point and target point including image-based visualization of the curved path at every level of trajectory.
- Kinematic model to determine and calculate the trajectories’ coordinates.
- Transformation of coordinates for the application on a stereotactic system.
- Availability of a mechanical system allowing the use of cannulas with different curves such as an actuation system for concentric tube robots or a system that allows the application of steerable needles.
- Availability of a CTCR prototype that allows the application of steerable needles of different curvatures in medical environments.

Considering the above, there is a gap between the methodology of stereotactic neurosurgical procedures. Various challenges arise regarding technical design and operation of a potential CTCR device, which enables (i) visual and technical planning of curved trajectories and (ii) application in a stereotactic procedure.

The authors present the first results of a joint project involving physicians, engineers, and mathematicians that aims at investigating the potential of stereotactic procedures with curved cannulas to reach a target point. In accordance with a clinical procedure, the study group used a path planning system for curved trajectories, a stereotactic system, and a cranial phantom that allowed a ‘pre-stereotactic’ CT-scan with stereotactic frame and localizers.

## Methods

### Path planning of curved trajectories

Based on previous works [16–20], we used an elastostatic model for representation of the CTCRs behavior presented by Rucker et al. [21]. The model includes interaction among the tubes as well as geometrical nonlinearity, yet neglects further mechanical phenomena like friction and material hysteresis. Path planning was performed on anonymized cranial 3 Tesla MR-images of patients, who have been treated in the authors’ department of neurosurgery for pathologic lesions where planning with a straight trajecory was expected to be difficult. Magnetization prepared rapid acquisition with gradient echoes (mprage)-sequences fused with diffuse tensor imaging (dti)-sequences for fiber identification of corticospinal and visual tract were used. The neurosurgeons of the research group identified and marked regions of interest and risk areas in each mprage slice. Based on the processed MR-image, mathematical constraints in form of target and entry region as well as obstacles were determined. In combination with CTCR model and tailored objectives, the path-planning problem was formulated, and an optimal cannula trajectory was derived numerically. The curved trajectory was verified by the neurosurgeons on the manual path planning software, who reviewed the curved path on axial, coronar and sagittal 2D-images as well as in a ‘probe’s eye view’.

In the present study, a concentric tube continuum robot (CTCR) platform was developed in several steps. A refined model was eventually fixed to a Riechert-Mundinger (RM) stereotactic device.

**Fig. 2:**
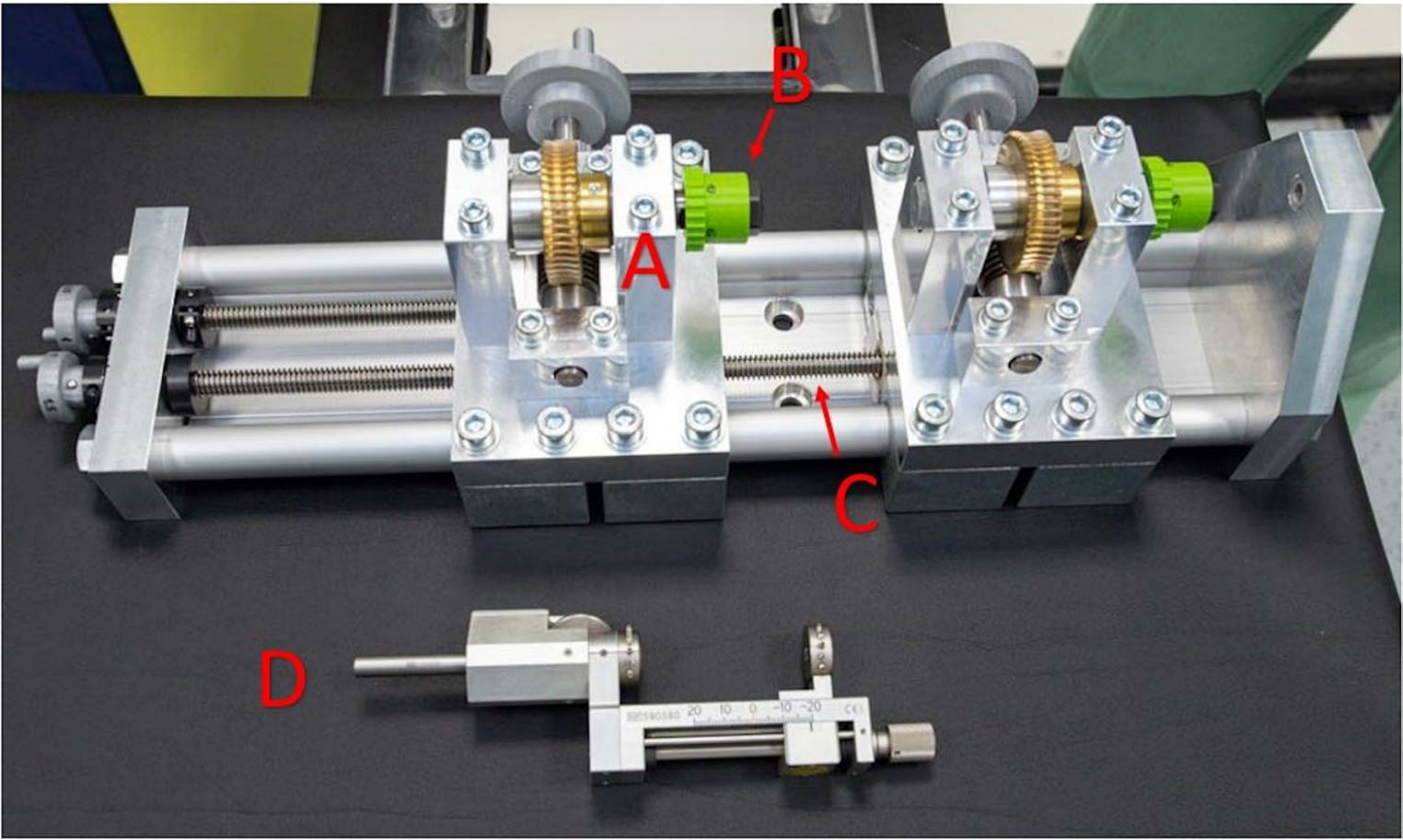
CTCR prototype and its components. Manually operated prototype of actuation system for extension and retraction of curved cannulas (**A**: bearing housings **B**: Collet for cannulas **C**: guide rails including spindle mechanisms and rotational drive). The elastostatic cannulas are deployed in the collets allowing translational and rotational movement. With the prototye, contact phenomena, friction and hysteresis were investigated to improve target point precision prior to its application with the stereotactic system. The stereotactic microdrive for micro-macro electrodes (**D**; Inomed, Emmendingen, Germany) at the bottom of the image illustrates the considerable size of the CRT prototype

At the beginning of the study, a CTCR platform (Fig. 2) that served as prototype was built to deploy shape-memory curved cannulas. Cannulas were made of nickel-titanium. It consists of a manually operated high-precision actuation apparatus and a photogrammetric system with measurement errors in the range of 100 μm. The platform was used to analyze target precision and ‘follow-the-leader-behavior’, meaning lateral movements during extension and retraction of the cannulas, *ex-situ*. The platform’s curved cannulas are composed of different sets of precurved cannulas with ascending diameters. Concerning its shape, each cannula was actuated translationally and rotationally at its base inside an actuation system. Due to the elastostatic behavior of the cannulas, contact phenomena, friction, and material hysteresis was investigated to improve target point precision. Repeated translational and rotational movements of the tubes and precise movement of the actuation apparatus were evaluated.

The prototype platform could not be attached to the aiming arm of the stereotactic system. Regarding positional accuracy of the CTCR with desired target deviations below 1 mm, a photogrammetric system including a contactless stereoscopic camera system with global shutter sensor for measurement of moving objects was used.

**Fig. 3:**
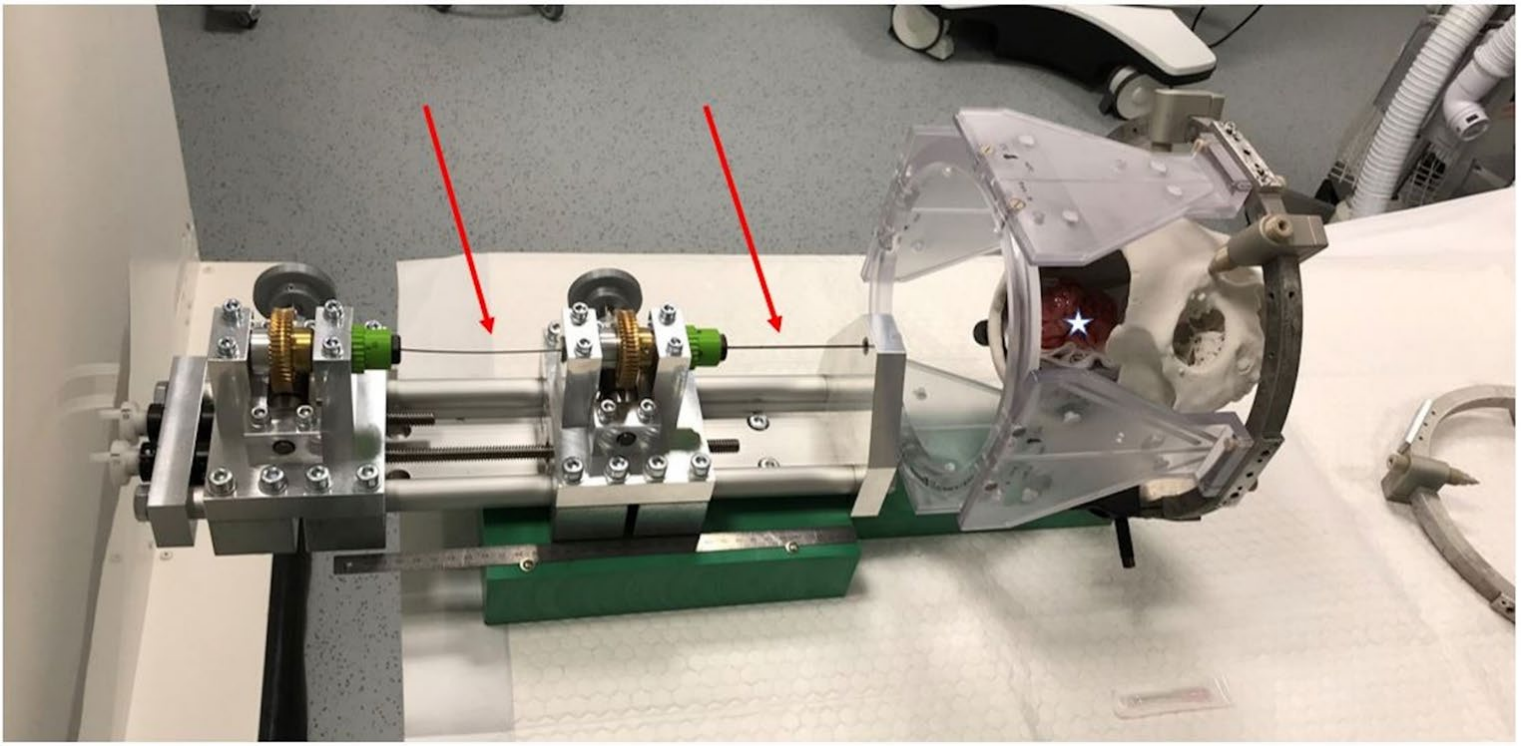
CTCR prototype used on porcine brain cadaver located in a cranial phantom. CTCR prototype (blue arrow) with the curved cannulas (red arrow) already fixed at the base of the actuator system. The cannulas are inserted into porcine brain cadavers, which was placed in a cranial phantom. The phantom was attached to the head frame of the RM stereotactic system. A CT-scan was performed in this configuration to evaluation metal artifacts

For a detailed description of the continuum robot research platform that was applied the authors refer to prior publications [17, 22, 23]. Potential interfering artifacts of CTCR and curved cannulas were assessed in a computed tomography (CT) scanner (Siemens Somatome Scope, Germany). Curved cannulas were inserted in a cranial phantom (3D printed plaster skull) fixed in the RM headframe, and in porcine brain cadavers (Fig. 3).

**Fig. 4:**
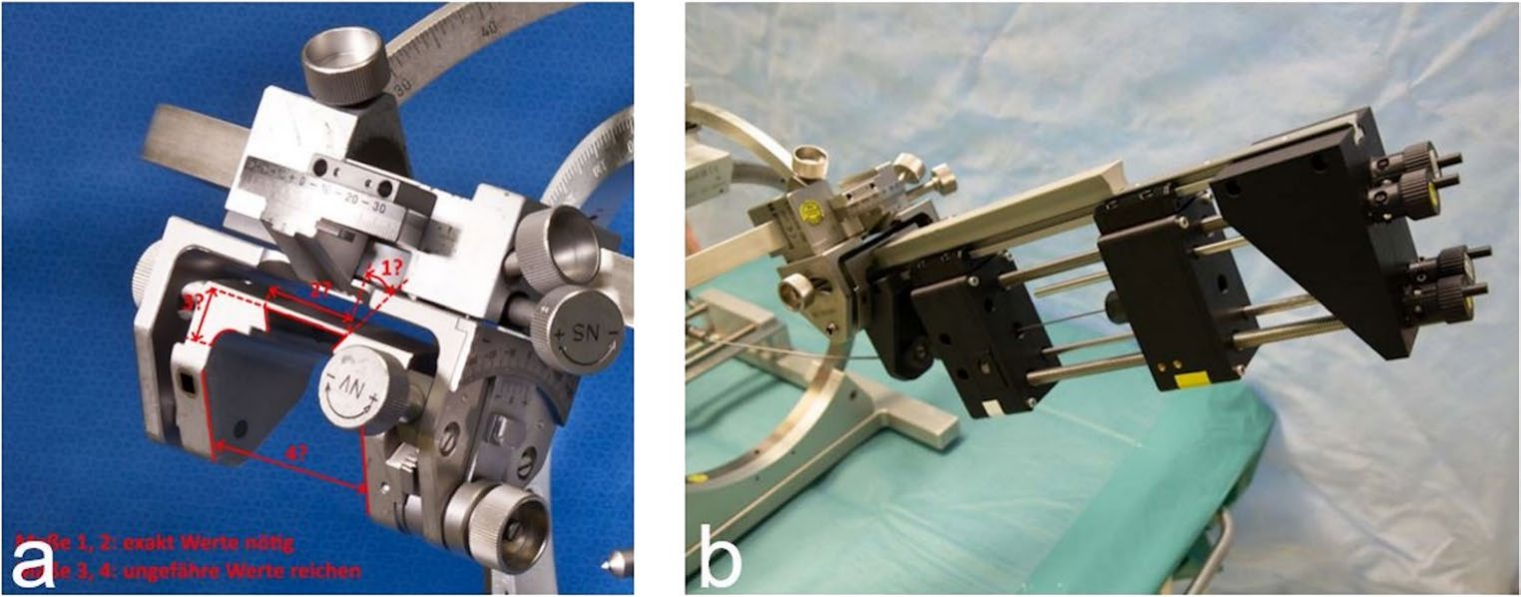
Connection of CTCR and RM stereotactic system. To attach the actuator system to the stereotaxic system, a dovetail was built (**A**). The dovetail was firmly connected to the actuator system’s guide for linear movement of both cannulas (**B**). Each cannula was deployed in a module for extension and retraction (**C**); the modules enable translational and rotational movement. Manual operation of the system is carried out with the hand wheels (**D**)

Attention was given to metal artifacts in the CT-scan of the actuation system and the curved cannulas while positioned within the cranial phantom and the porcine brain parenchyma. Lastly, the actuation system was modified for fixation at the aiming arm unit of the RM stereotactic system (Fig. 4).

### Determination of the ctcr’s actuation variables

To enable movement of the curved cannulas out of the actuation system to the defined target, determination of translational und rotational setting parameters for the cannulas is needed. By attaching the actuation system to the RM aiming arm unit, the RM stereotactic system and its polar coordinates were utilized for setting the CTCR’s location and pose near the cranial phantom. The necessary RM-parameters were calculated after the CTCR’s pose was determined by path planning according to the geometric relations of the RM-system [24].

### Stereotactic procedure including standardized cranial Phantom

To evaluate if a stereotactic procedure using the CTCR is in general possible in general, the developed hardware and software components of the different working groups were connected and tested in a procedure resembling an intracranial stereotactic biopsy. For this part, 3D printed plaster skulls (Zimmer-Biomet, Eschbach, Germany) were prepared for the stereotactic CT-scan. In accordance with Krüger et al. [25], a model with radiological characteristics similar to those of a human skull was made. After preparation with plaster stripes and varnish, the skull was filled with 10% agar. Radiopaque targets of different sizes made of bone cement (Palacos^®^, Hareus Medical GmbH, Wehrheim) were placed in different locations within the skull. Preparation of the cranial phantoms was done prior to the stereotactic experiment; the phantoms were found to be storable at a temperature of 8 °C for several weeks.

The cranial phantoms were positively (0°) fixed in the RM base ring and localizers were attached. After a 2-mm CT-scan, the target was chosen and data was sent to the department’s neurosurgical planning station (Medtronic StealthStation S7, Meerbusch, Germany). Polar coordinates for a straight trajectory to the target were obtained for the RM stereotactic system. Computer-tomographic images and RM-coordinates were delivered to the study groups’ path planning system, and a curved path was planned based on image recognition. Translational und rotational setting parameters of the CTCR were obtained. The aiming arm unit including the actuator system was attached to the target point simulator. The precision of the curved trajectory was verified by extending the CTCR’s cannulas according to the planned actuation parameters. Lastly, the cannulas were set back, and the aiming arm was mounted at the base ring. A 14 mm burr hole trepanation was performed at the planned entry point, and the cannula was driven out of the actuation system. A second CT-scan was performed to identify the tip position of the curved cannula. Both CT-scans were fused at the neurosurgical planning station to exclude movement of the target and to compare the final target point with the calculated (polar) target point.

## Results

### Path planning of curved trajectories

For planning of the curved trajectories using the developed mathematical toolchain [19], MRI-data of patients, who were treated surgically for intra-axial lesions in the authors’ department between 03/2020 and 07/2022 were evaluated. Out of 561 identified surgeries, data including MPRAGE- and dti-sequences were considered for curved path planning, when targeting with a straight path was considered likely to be difficult (i.e. located in the insular region, basal ganglia, and pineal region/posterior midbrain). Out of 90 identified temporal lesions, 14 lesions were located at the insula. Further, data sets from 16 patients with lesions within the basal ganglia region, and seven patients with lesions in the dorsal midbrain were examined in more detail. Eventually, 10 datasets from patients were selected.

**Fig. 5:**
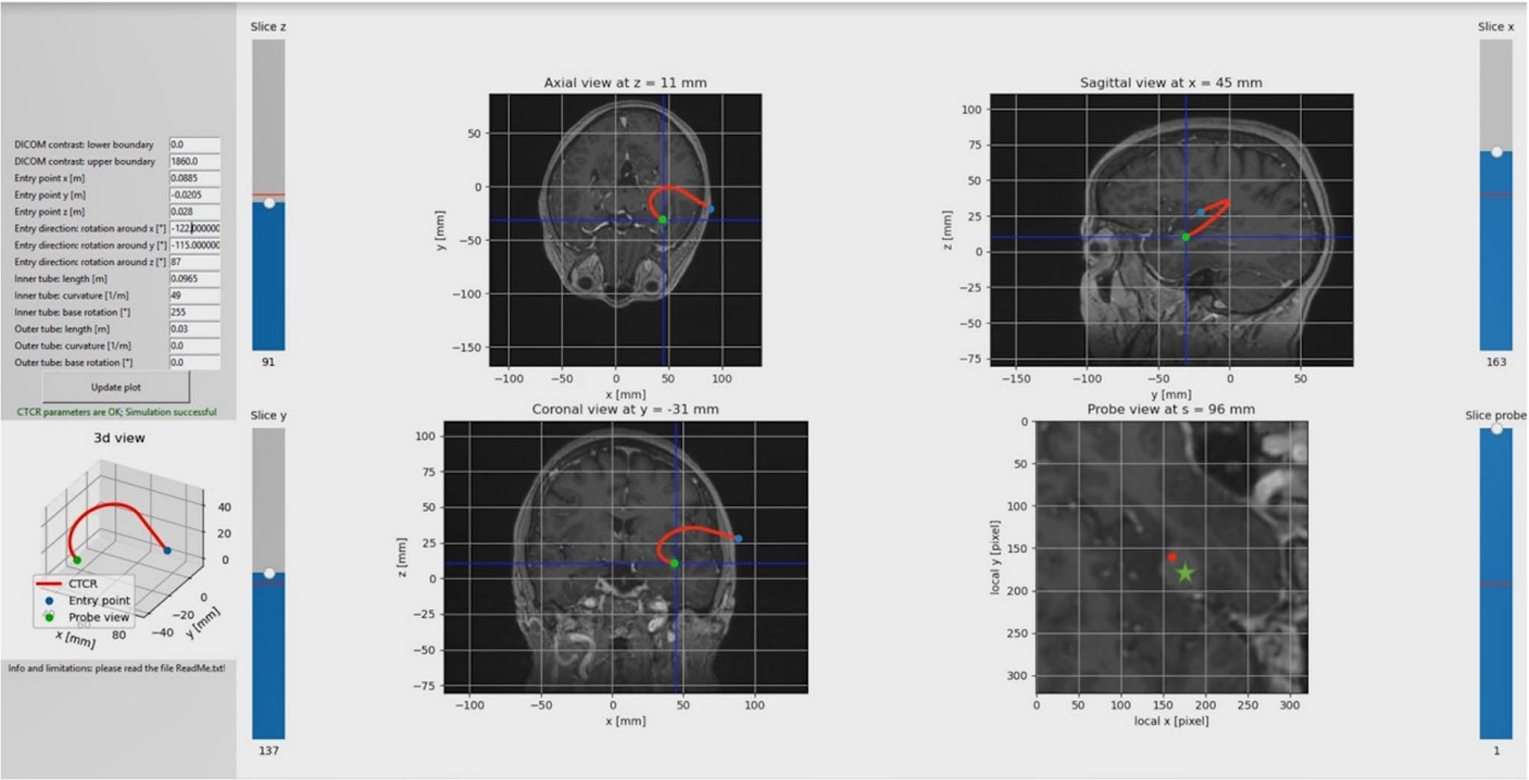
Planning of curved path on MRI-data of a patient with a lesion located in the insular region. Path planning of a curved trajectory. A lesion located at the insular region with weak contrast enhancement (green asterisk) was targeted. Path planning with axial, sagittal and coronar view was difficult. Potential risk factors for complications such as arteries and sulci could be best assessed with the‚ probe’s eye view‘

In summary, path planning with the developed algorithms was possible, but very time consuming (Fig. 5). Planning included the marking of ‘prohibited’ cerebral areas in every slice of the MPRAGE-sequence such as primary cortex, sulci, vessels, cortico-spinal tract, and basal ganglia, which took up to two days per dataset. Further, unlike with straight trajectories, the expected path is difficult to estimate with the manual path planning tool [14], which resulted in many path corrections that took a considerate amount of time. Lastly, it must be noted that in the present experimental setup the manual path planning is restricted to the usage of two cannulas for reduced complexity. Thus, the possible CTCR’s shape is limited when compared to using three tubes.

**Fig. 6:**
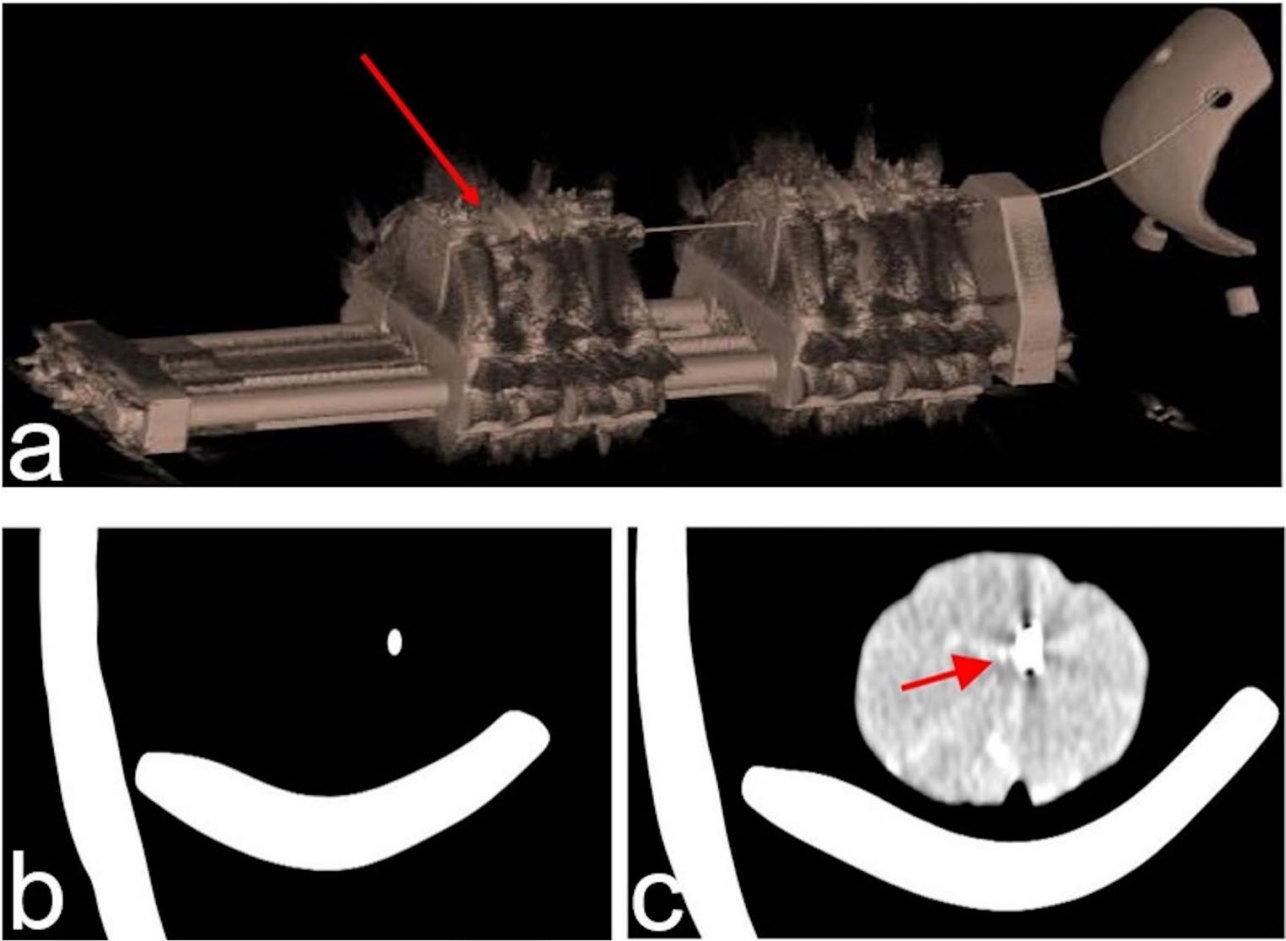
Analysis of metal artifacts of the CTCR. Connection of CTCR and RM stereotactic system. Analysis of CT-scans revealed moderate metal artifacts (arrows) of the actuation system as well as the cannulas within the cranial phantom and the porcine brain cadavers. Artifacts mainly occurred around the actuation modules (**a**). The cannulas‘ artifacts were less in the cranial phantom (**b**) compared to porcine brain tissue (**c**)

For further testing of the accuracy and applicability of the CTCR’s actuation system when using it in combination with the stereotactic system, the CTCR as well as the stereotactic headframe were mounted on common basement. Particular attention was given to X-ray artifacts of the actuation system and curved cannulas during CT-scan. Artifacts were moderate and mainly found around the actuation modules. At the base ring, artifacts were seen as expected from routine clinical applications (Fig. 6a). Image quality at the region of interest did not show significant artifacts. At the head frame, artifacts were seen as expected from routine clinical applications. For evaluation of artifacts within brain parenchyma, porcine brain was placed within 3D cranial phantom without agar that was fixed at the head frame. The curved cannula was inserted into the porcine brain, and CT-scans were performed to identify the conduct of the curved cannula within the brain parenchyma. The images did not show marked tearing of the brain parenchyma. However, a higher degree of interfering cannula artifacts was observed within the parenchyma compared to the cranial phantom. Artifacts depended on the amount of porcine cadaver tissue and on the angle of X-rays on the cannula (Fig. 6b).

### Stereotactic procedure with standardized cranial Phantom

**Fig. 7:**
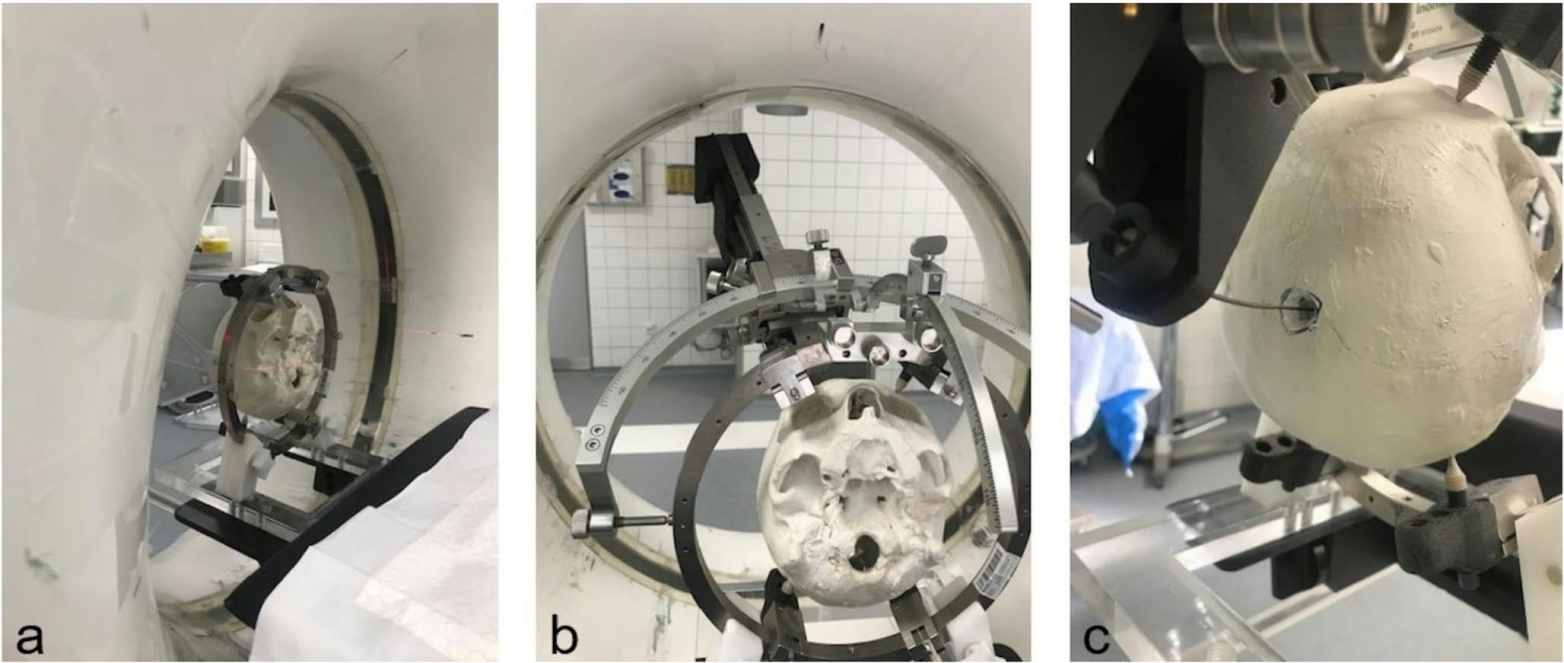
Stereotactic procedure with application of the CTCR in a cranial phantom. After verification of target precision at the target point simulator, the aiming arm was mounted at the base ring (**b**). A 14 mm burr hole trepanation was performed at the entry point, and the cannulas were moved out of the actuation system a second time (**c**), before a second CT-scan was performed to identify the tip of the curved cannula

**Fig. 8:**
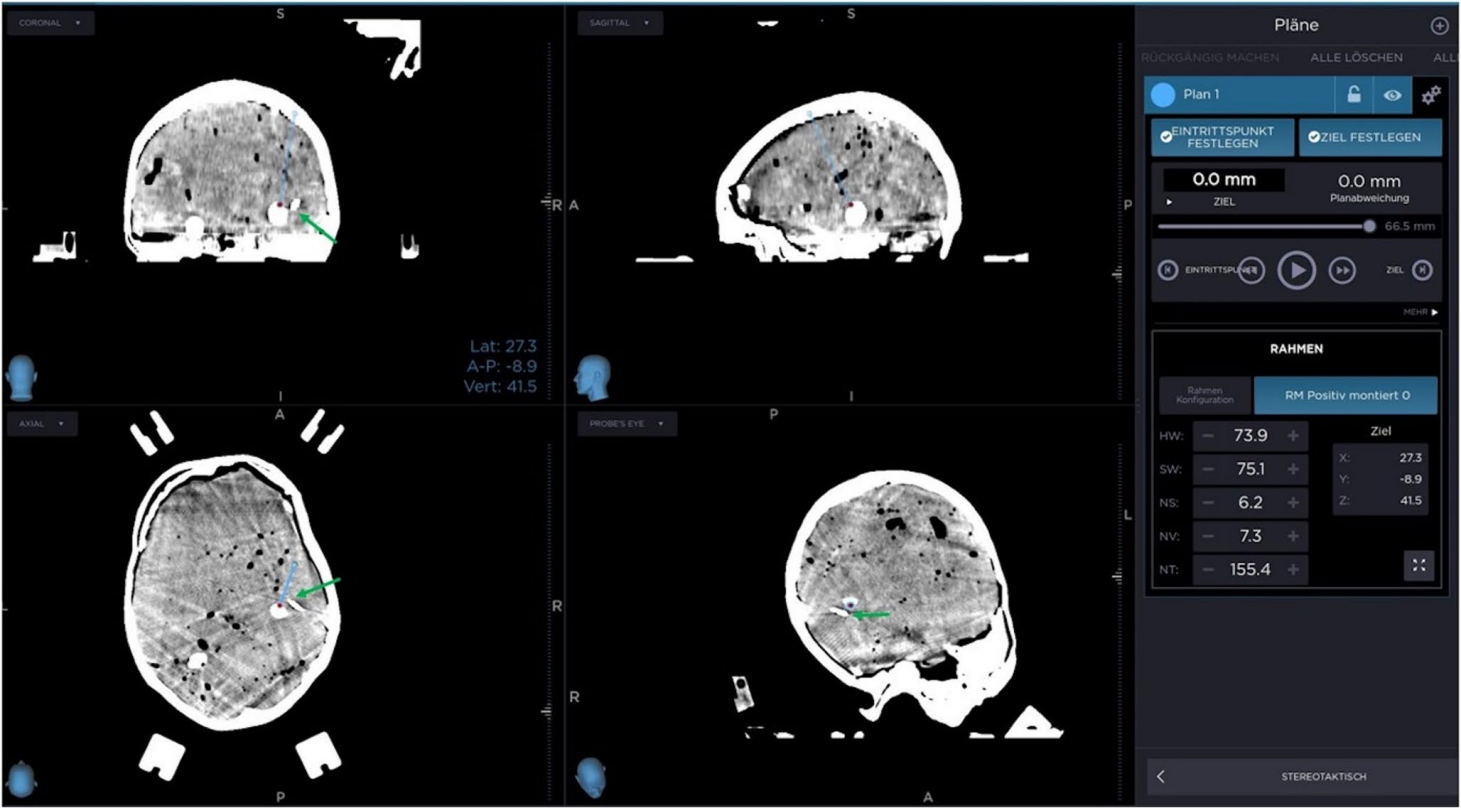
Comparison of planned and final position of target point. Comparison of the final position of the curved cannulas tip (green arrow) and the tip of the (virtual) target point in a straight trajectory (red dot/blue trajectory). Despite target point accuracy in the numerical optimization model, the curved cannulas hat a target point deviation of > 2 mm, which is higher than allowed

For further experiments in the OR, the CTCR prototype was adapted to be mountable at the RM stereotactic system. The stereotactic systems’ dovetail was copied, manufactured, and attached to the CTCR. Thus, it was possible to use the CRCT system in a way that allows further evaluation, if a stereotactic procedure with curved cannulas is feasible in a ‘clinical set-up”. A stereotactic procedure using the 3D cranial phantom was possible (Fig. 7). However, despite optimal target point accuracy in the planned configurations by numerical optimization, the first practical applications of curved cannulas had a target point deviation of > 2 mm (4–8 mm), which is far higher than allowed (Fig. 8).

An analysis of all individual process steps showed that a large part of the total deviation is resulting from the used kinematic model for simulating the CTCR’s behavior, which is – as stated above – not able to cover material nonlinearity, material hysteresis, and friction. In separate tests, where path planning was done with a single straight tube being inserted into the CTCR, target deviations were below 1 mm, proving that all the other components of the workflow – the path planning principle, RM-coordinate calculation, CTCR actuation system errors etc. – are not causing significant errors larger than in surgical practice and are, thus, valid.

## Discussion

Today’s stereotactic procedures are based on surgical instruments with straight cannulas. Over the past years, various research groups from different disciplines have investigated cannulas with adjustable curvatures. Curved paths open up possibilities for additional interventional scenarios and could significantly reduce the surgical risk.

In everyday clinical practice, physicians use instruments that were developed in the 1950 s and adapted over the decades to modern materials and technical improvements such as neuronavigation [26, 27].

If the targeted pathology is not accessible in a safe way during stereotactic surgery, the procedure cannot be performed as planned. Either a second-choice trajectory or a different surgical technique (e.g. an open, more invasive procedure) is considered. The alternative is often associated with a higher risk for complications. With increasing depth and distance, the number of potentially eloquent, sensitive brain areas increases.

The use of a system with a curved cannula would offer a multitude of additional planning options for reaching a target with minimal surgical risk while bypassing functionally important fiber tracts or blood vessels. So far, published papers do mainly address questions of robot design, robot control, and path planning. Medical experiments imitating a stereotactic (intracranial) procedure are missing. Currently, different research groups are working on the development of curved cannulas specifically designed for medical treatment.

There are various approaches to solve the problem of how to integrate curved instruments into medical interventions and surgical procedures. Most studies focus on minimally invasive surgical techniques in disciplines other than neurosurgery. Over the past years, few studies have been published that address the possible use of curved pathways in the field of neurosurgery [5–7, 9]. Several studies emphasize future techniques for epilepsy surgery and techniques for intracranial endoscopy [8, 9]. Kim et al. presented an intracranial mini-robot for brain tumor diagnosis and therapy [9]. An intention to use these developments for intracerebral stereotactic application was not described. An interdisciplinary research group of the EU-funded Horizon project ‘Enhanced Delivery Ecosystem for Neurosurgery 2020’ (EDEN2020) worked on the development of a ‘treatment system for one-stop diagnosis and minimally invasive treatment in neurosurgery’. They published several results regarding minimally invasive surgical and endoscopic applications of various medical disciplines [28–31]. As part of the project, researchers investigated a model for 3D steering of so called programmable bevel-tip needles – a different type of continuum robot than CTCRs – inspired by the biological model of parasitic wasps’ ovipositors [31]. In comparison with CTCRs, the programmable bevel-tip needle offers extended maneuverability, while, on the other hand, the mechanical principle as well as control of the tip position are far more complex, and thus, questions regarding medical safety are difficult to assure. Also, the design principle of this type of active cannula is based on interacting forces between brain matter and the cannula, since the bending stiffness of the cannula backbone is low. A literature search in the medical database yielded no results regarding the use of this technique in neurosurgical stereotactic procedures. To the best of our knowledge, there is currently no physical system in preclinical or clinical testing that represents a stereotactic procedure comparable to everyday clinical practice, i.e. translation of straight trajectories to curved trajectories and transformation of these coordinates to a stereotactic system in the field of neurosurgery.

In the past decades, different path planning algorithms for curved trajectories have been developed. Research included sampling-based and potential field-based algorithms as well as algorithms using artificial intelligence (AI); [32, 33]. Special attention was given to three main approaches: path planning with straight trajectories, curved path planning with steerable needles, and curved path planning with concentric tubes [32]. Most publications focus on automated path planning for straight trajectories, which aims at improving the safety of stereotactic procedures as well lowering time consumption for trajectory planning [32, 33, 38].

While the use of straight trajectories is already part of clinical practice, research groups emphasized that curved trajectories are currently studied in different simulation models without any kind of clinical model [32, 34]. In general, they address the challenge of finding the optimal pathway for a specific type of curved cannula instrument subject to surgical constraints like, e.g., possible entry regions and eloquent areas of the brain. In neurosurgical context, different algorithms based on the ‘bevel-tip needle’ design including tests in simulation and 3D models of the brain were published [3, 30, 31].

Regarding intracranial stereotactic procedures, Segato et al. investigated an algorithm for deep brain stimulation (DBS). In MR-images of 10 volunteers, a physician marked the cortical entry point and subthalamic nucleus (STN) as target point. Fusion with dti-sequences for planning of the corticospinal tract was performed. The physician’s straight trajectories were compared to algorithm-based curved trajectories. Further, a research group of the EDEN2020 project presented a path planning approach for different 3D curved paths. They used a virtual 3D cranial image obtained from an anonymized MR-image data set. Their goal was to develop technology for convection enhanced drug delivery for cancer treatment using a steerable needle [35]. However, the studies were computational without translation and transfer of the automated curved trajectories to a stereotactic system or ‘into the OR’ [3, 35].

Dependent on the surgical techniques that require an insertion of curved cannulas as part of the treatment, different models have been investigated over the past years [5, 6, 21, 32]. Operating the CTCRs kinematic constraints while avoiding eloquent structures is challenging. Neuronavigation registration inaccuracies, brain shift during surgery, and mechanical inaccuracies must be considered. Alfalahi et al. published their results on the potential application of CTCRs in different surgical specialties and proposed less traumatic surgical approaches and manipulation [36]. They considered the treatment of hydrocephalus, intracerebral hemorrhage, epilepsy and pituitary gland tumors. An approach in terms of preclinical or clinical study was not addressed. Other research groups with emphasis on neurosurgical procedures with the aid of CTCRs focus on minimally invasive techniques for epilepsy surgery [5, 8, 37], intracranial endoscopy [9], and brain tumor diagnosis and therapy [7]. Frisken et al., who developed a risk map based on probabilistic segmented uncertainty zones in cranial MR-images, proposed that sources of uncertainties in CTCR path planning are handled primarily by adding a ‘safety margin’ of 2–3 mm around the critical structures to avoid penetration through these structures. However, even if a safety margin is considered, the CTCRs hysteresis effects oftentimes not being accounted for in path planning increase with length and number of tubes, which can lead to distortion of brain parenchyma and disruption of vessels.

## Limitations of the study

In the present study, we focused on CTCRs for curved trajectory guidance with a cannula mechanism consisting of nested nickel-titanium tubes, which in our view has the greatest potential for use in stereotactic neurosurgery due to its superelastic properties and its long time use in the medical field. The mechanisms high flexural rigidity, slim structure, actuation outside the cannula, potential to generate precise trajectory planning and approximate follow-the-leader behavior are promising properties. At present, we had deviations between 2 mm and 8 mm in the clinical set-up. There are various reasons for the deviation between the final and calculated target point. As described above, several technical challenges including hysteresis effects, design of preformed elastic tubes, and their sensitivity to manufacturing tolerances occur. This influences the follow-the-leader behavior and impairs target point accuracy. Considering stereotactic procedures, precision is of utmost importance. In the authors’ view, a ‘safety margin’ of 2–3 mm might even result in a reduced precision at the target point, which might only be 5 mm in diameter in a stereotactic procedure.

Another challenge is the current size of the CTCR’s actuation system. For now, the actuation system used in this study is too large and too heavy for the use in a conventional stereotactic procedure. Due to the systems length, the aiming arm units’ applicability concerning freedom of movement is impaired. Further, with the present actuation system, the use of only two nested tubes is currently possible. To reach any location within the skull model, to obtain such a trajectory with a CTCR system, three nested cannulas would be ideal.

Another important aspect is the use of tube materials with special memory characteristics. In the current CTCR actuation system, separation of the actuator system’s collet and cannulas is not possible, as this ensures the calculated curved trajectory. Thus, removal of the collet would currently change the calculated path. However, for the passage of instruments such as biopsy devices or even implants (e.g. electrodes), decoupling of the collet and cannulas is necessary. Therefore, the development of such cannulas and instruments is of importance and must be considered in future studies.

Regarding a faster path planning, the research group aims to develop a trajectory-planning algorithm in the sense of an AI-based ‘a-priori’- adaptation in further work. Additionally, studies demonstrating the curved tubes’ impact on biologic tissue such as disruption in various areas of the brain, including grey and white matter, as well as injury to larger and smaller vessels are missing. Thus, further medical experiments including histological evaluation are intended.

## Conclusion

Considering the difficulties that are yet to be solved, to the authors’ knowledge, this is the first model demonstrating the application of curved pathways imitating a stereotactic procedure including CT-based target planning, coordinate calculation and transformation, and application of a system for curved cannulas in a head model. Over the course of the experiments, CTCR platform was re-designed to improve mechanical properties to enable it to reach every area within the skull. The mechanical design included an aiming arm adapter for attaching the actuation system to the stereotactic system. Additionally, a planning tool for curved trajectories has been developed. Mathematicians and engineers have designed and implemented an algorithm for converting the CTCR’s path planning result into coordinates for the stereotactic aiming arm. Finally, the target point of the CTCR was tested in the neurosurgical OR as a ‘stereotactic procedure’.

## Data Availability

No datasets were generated or analysed during the current study.

## Abbreviations

AI: Artificial intelligence
CT: Computertomography
CTCR: Concentric tube robot / concentric tube continuum robot
DBS: Deep brain stimulation
dti: Diffusor tension imaging
EU: European Union
Mm: Millimeter
mprage: Magnetization prepared rapid acquisition with gradient echoes
MRI: Magnet resonance tomography
OR: Operating room
RM: Riechert Mundinger
STN: Subthalamic nucleus
3D: Three-dimensional
